# Random forest differentiation of *Escherichia coli* in elderly sepsis using biomarkers and infectious sites

**DOI:** 10.1038/s41598-024-63944-6

**Published:** 2024-06-05

**Authors:** Bu-Ren Li, Ying Zhuo, Ying-Ying Jiang, Shi-Yan Zhang

**Affiliations:** Department of Clinical Laboratory, Fuding Hospital, Fujian University of Traditional Chinese Medicine, 120 South Road of Old City, Fuding, 355200 Fujian China

**Keywords:** Sepsis, Elderly, Machine learning, Random forest, *Escherichia coli*, Diagnostic accuracy, Biomarkers, Diseases, Medical research

## Abstract

This study addresses the challenge of accurately diagnosing sepsis subtypes in elderly patients, particularly distinguishing between *Escherichia coli (E. coli)* and non-*E. coli* infections. Utilizing machine learning, we conducted a retrospective analysis of 119 elderly sepsis patients, employing a random forest model to evaluate clinical biomarkers and infection sites. The model demonstrated high diagnostic accuracy, with an overall accuracy of 87.5%, and impressive precision and recall rates of 93.3% and 87.5%, respectively. It identified infection sites, platelet distribution width, reduced platelet count, and procalcitonin levels as key predictors. The model achieved an F1 Score of 90.3% and an area under the receiver operating characteristic curve of 88.0%, effectively differentiating between sepsis subtypes. Similarly, logistic regression and least absolute shrinkage and selection operator analysis underscored the significance of infectious sites. This methodology shows promise for enhancing elderly sepsis diagnosis and contributing to the advancement of precision medicine in the field of infectious diseases.

## Introduction

Sepsis is a significant healthcare challenge, especially for the elderly. It results from an overactive immune response to infection, which can lead to life-threatening organ dysfunction^1^. The diverse clinical presentations and causative agents of sepsis underscore the need for precise diagnosis in elderly sepsis patients. Notably, *Escherichia coli* (*E. coli*), a prominent Gram-negative bacterium, plays a pivotal role in this scenario. Known for inducing intra-abdominal and urinary tract infections^2^, *E. coli* often escalates to sepsis, exacerbating the severity of the condition^3^. Recognized globally as a prevalent pathogen, *E. coli* frequently contributes to both bloodstream and urinary tract infections^4–6^. Given its substantial impact, tailored management approaches for *E. coli*-related sepsis are indispensable for ensuring effective diagnostic protocols, particularly in the elderly population^7^.

Biomarkers play a crucial role in sepsis diagnostics and management, offering insights into underlying mechanisms and aiding in patient categorization for tailored treatments^8^. Traditional biomarkers such as white blood cell count (WBC), C-reactive protein (CRP), and procalcitonin (PCT) have been widely used for diagnosing sepsis^9^. However, their effectiveness varies in terms of specificity and sensitivity, often falling short in providing a comprehensive assessment of the sepsis syndrome, particularly in distinguishing between bacterial strains.

Integrating machine learning into healthcare signifies a transformative shift towards more accurate diagnostics and enhanced patient outcomes^10^. Models like the random forest algorithm excel in dissecting complex clinical datasets, discerning patterns, and forecasting clinical scenarios with greater accuracy than conventional statistical approaches^11^. Such models adeptly merge diverse clinical indicators and biomarkers, fostering a deeper comprehension of multifaceted diseases, including sepsis^12^. Sepsis is a complex syndrome encompassing a spectrum of clinical manifestations, making accurate diagnosis challenging. *E. coli*, a subgroup of bacteria known for their diverse pathogenicity, has been implicated in sepsis cases. However, the identification of biomarkers, infectious locations, and the factors contributing to their presence in elderly sepsis patients with *E. coli* infection has not been fully explored.

To bridge this knowledge gap, we conducted a retrospective observational study on elderly sepsis patients, both with and without *E. coli* infections. The study leveraged a multidimensional dataset comprising demographic information, comorbidities, hematological parameters, and details of the infection site. Our investigation involved a rigorous statistical analysis, machine learning techniques, and feature importance analysis to identify the key predictors of biomarkers and infectious sites in sepsis elderly patients with and without *E. coli* infection.

## Results

### Baseline characteristics of participants

This study included 119 consecutive hospitalized elderly patients, divided into an *E. coli* sepsis group (n = 57) and a non-*E. coli* sepsis group (n = 62). Table 1 summarizes the baseline characteristics of the participants, analyzed statistically using the CBCgrps Package in R^13^. The median age was 73 years, with a slightly higher proportion of females (53%) than males (47%). A majority (92%) of participants abstained from drinking, and 90% did not smoke. The most common comorbidities were hypertension (69%) and cardiovascular diseases (10%), with 38% of participants having diabetes. Laboratory measurements such as hemoglobin (HGB), mean corpuscular volume (MCV), red cell distribution width (RDW), white blood cell count (WBC), neutrophil, lymphocyte, monocyte, platelet count, platelet distribution width (PDW), mean platelet volume (MPV), C-reactive protein (CRP), procalcitonin (PCT), triglycerides (TG), cholesterol, uric, albumin (ALB), albumin-CRP ratio (ALB_CRP), and others were recorded. Significantly, the only notable difference between the *E. coli* and non-*E. coli* sepsis groups was in HGB levels (*P* = 0.036).

**Table 1:** Baseline characteristics of participants in the study. Continuous variables were presented as median (Q1, Q3) for skewed data or mean ± SD for normally distributed data. Categorical variables were presented as n (%). Q1, Q3, first and third quartiles, respectively; n (%), Number of participants and percentage; HGB, hemoglobin; MCV, mean corpuscular volume; RDW, red cell distribution width; WBC, white blood cell count; PDW, platelet distribution width; MPV, mean platelet volume; CRP, C-reactive protein; PCT, procalcitonin; TG, triglycerides; ALB, Albumin; ALB_CRP, albumin-CRP ratio; SD, standard deviation; Abdominal, infections located in the abdominal area; Pulmonary, infections located in the lungs; Urinary, infections located in the urinary tract; Other, infections located in areas not specified above.

Variables	Total (n = 119)	Non-E. coli (n = 62)	coli (n = 57)	*P* value
Gender, n (%)				0.222
Female	63 (53)	29 (47)	34 (60)	
Male	56 (47)	33 (53)	23 (40)	
Drinking, n (%)				0.494
No	110 (92)	56 (90)	54 (95)	
Yes	9 (8)	6 (10)	3 (5)	
Smoking, n (%)				0.01
No	107 (90)	51 (82)	56 (98)	
Yes	12 (10)	11 (18)	1 (2)	
Hypertension, n (%)				0.378
No	37 (31)	22 (35)	15 (26)	
Yes	82 (69)	40 (65)	42 (74)	
Cardiovascular, n (%)				0.647
No	107 (90)	57 (92)	50 (88)	
Yes	12 (10)	5 (8)	7 (12)	
Diabetes, n (%)				0.265
No	74 (62)	42 (68)	32 (56)	
Yes	45 (38)	20 (32)	25 (44)	
Age (year)	73 (69, 79)	73 (69, 78)	76 (70, 81)	0.235
HGB (g/L)	110 (91, 119)	112 (100.75, 123)	109 (88, 115)	0.036
MCV (fL)	89.1 (85.3, 93.1)	89.16 (85.85, 93.4)	88.5 (84.8, 92.9)	0.613
RDW (%)	13.7 (13, 14.6)	13.6 (12.93, 14.48)	13.7 (13, 14.8)	0.89
WBC (× 10^9^/L)	10.12 (7.36, 16.21)	10.18 (7.23, 16.29)	10.02 (7.53, 15.87)	0.796
Neutrophil (× 10^9^/L)	8.49 (5.71, 14.71)	8.46 (5.73, 14.79)	8.49 (5.75, 13.7)	0.724
Lymphocyte (× 10^9^/L)	0.99 (0.57, 1.31)	0.85 (0.55, 1.17)	1.13 (0.62, 1.31)	0.154
Monocyte (× 10^9^/L)	0.49 (0.33, 0.66)	0.5 (0.32, 0.66)	0.48 (0.38, 0.68)	0.651
Platelet (× 10^9^/L)	161 (115, 214.5)	161 (100, 205.06)	157 (126, 237)	0.182
PDW (%)	11.4 (10.3, 13.05)	11.52 (10.1, 13.88)	11.2 (10.3, 11.9)	0.381
MPV (fL)	10.42 (9.95, 11.4)	10.5 (10, 11.47)	10.4 (9.9, 11.3)	0.558
CRP (mg/L)	109.49 (61.44, 151)	107.04 (55.48, 151)	111.3 (64.08, 151)	0.836
PCT (ng/mL)	5.69 (0.84, 21.02)	3 (0.44, 20.91)	8.2 (2.79, 20.99)	0.183
Cholesterol (mmol/L)	3.53 (2.96, 3.53)	3.53 (3.02, 3.59)	3.53 (2.99, 3.53)	0.365
TG (mmol/L)	1.42 (1.13, 1.42)	1.42 (1.13, 1.42)	1.42 (1.15, 1.42)	0.421
Uric (umol/L)	262 (183, 337)	262.5 (206.5, 328.5)	262 (178, 339)	0.947
ALB (g/L)	32.07 ± 4.73	31.72 ± 4.9	32.47 ± 4.56	0.389
ALB_CRP	0.3 (0.21, 0.52)	0.3 (0.21, 0.53)	0.3 (0.21, 0.51)	0.896
Site, n (%)				< 0.001
Abdominal	24 (20)	10 (16)	14 (25)	
Pulmonary	36 (30)	30 (48)	6 (11)	
Urinary	38 (32)	6 (10)	32 (56)	
Other	21 (18)	16 (26)	5 (9)	

### Correlation analysis among various clinical biomarkers

**Figure 1:**
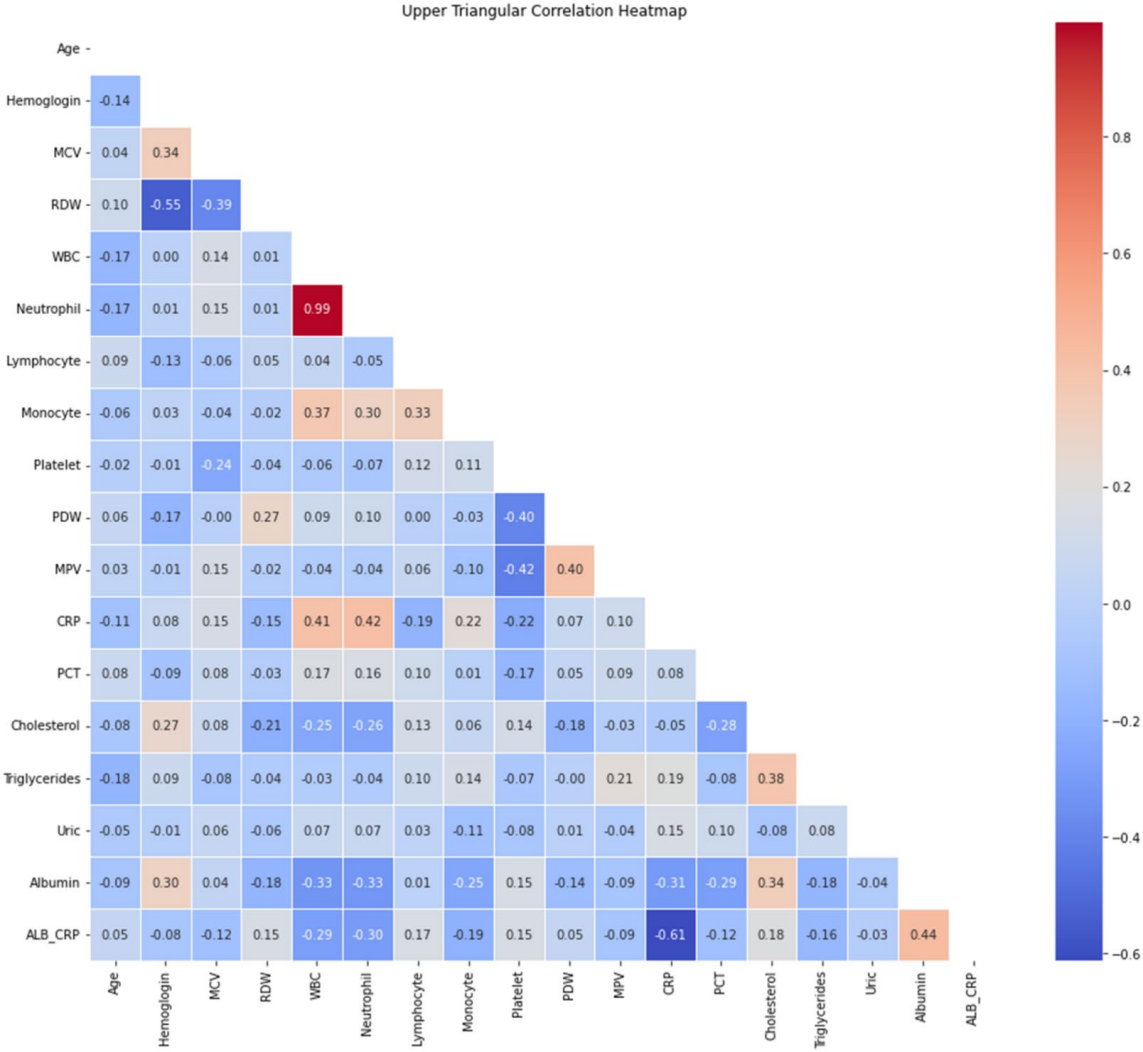
Pairwise correlation coefficients among 18 clinical biomarkers. The color gradient indicates the strength and direction of the correlations, with blue representing negative correlations and red representing positive correlations. HGB, hemoglobin; MCV, mean corpuscular volume; RDW, red cell distribution width; WBC, white blood cell count; PDW, platelet distribution width; MPV, mean platelet volume; CRP, C-reactive protein; PCT, procalcitonin; TG, triglycerides; ALB, albumin; ALB_CRP, albumin-CRP ratio.

To further simplify the predictive model, this study analyzed pairwise Spearman’s correlation coefficients (r) among 18 statistically significant biomarkers, excluding sex, through heatmap visualization. This approach helped identify and exclude highly correlated variables to streamline the model. The heatmap analysis, illustrated in Fig. 1, revealed the interrelationships between clinical biomarkers in elderly sepsis patients. A notably strong positive correlation (r = 0.99) between neutrophil counts and WBC indicated a close association between their levels. Conversely, a significant negative correlation was found between CRP levels and the ALB-CRP ratio (r = − 0.61), suggesting an inverse relationship in the context of sepsis progression. The correlations for the rest of the variables were relatively weak (absolute value of r < 0.6), emphasizing their distinct contributions to the disease process. These insights highlighted the importance of certain biomarkers in enhancing the diagnosis and management of sepsis among the elderly.

### Distribution of microorganisms

**Figure 2:**
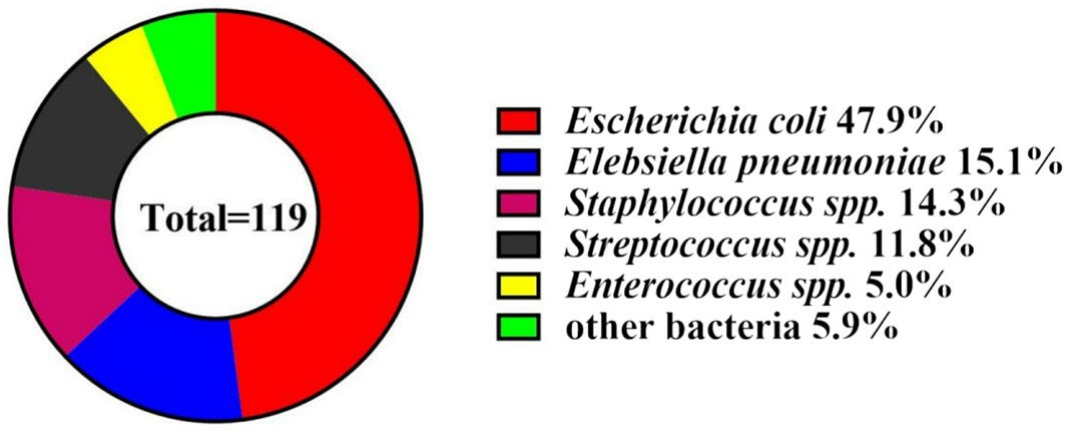
Bacterial pathogen distribution in elderly sepsis patients (n = 119 isolates). The doughnut chart shows the prevalence of different bacteria in the study, with *Escherichia coli* being the most common (47.9%), followed by *Klebsiella pneumoniae* (15.1%), *Staphylococcus *spp. (14.3%), *Streptococcus *spp. (11.8%), *Enterococcus *spp. (5.0%), and other bacteria (5.9%).

The study identified 119 bacterial strains in elderly sepsis patients, comprising 37 Gram-positive (31.1%) and 82 Gram-negative (68.9%) strains. *E. coli*, with 57 strains (47.9%), was the most prevalent, followed by *Klebsiella pneumoniae* at 18 strains (15.1%), highlighting the dominance of Gram-negative bacteria in sepsis cases. Additionally, *Staphylococcus spp*. (17 strains, 14.3%), *Streptococcus spp*. (14 strains, 11.8%), and *Enterococcus spp*. (6 strains, 5.0%) were significant, demonstrating the microbiological diversity impacting sepsis diagnostic strategies. This diversity underscored the necessity for broad-spectrum empirical diagnoses to address the various potential bacterial causes of sepsis (Fig. 2).

### Logistic regression analysis

Tables 2 and 3 display the results of both univariate and multivariate logistic regression analyses. The results of univariate logistic regression analysis showed that smoking (*P* = 0.019), the infectious site of abdominal (*P* = 0.023) and urinary (*P* < 0.001) were significant factors (Table 2). Nevertheless, in the multivariate logistic regression analysis, the variable of smoking showed no statistically significant (*P* = 0.157). The infectious site of urinary emerged as a significant factor in both univariate and multivariate logistic regression analyses (*P* < 0.001), with an odds ratio (OR) of 14.380 (95% confidence intervals [CIs]: 3.552, 58.207) in the multivariate logistic regression model, indicating a strong association with the type of infection.

**Table 2:** Univariate logistic regression analyses results for clinical feature. B; coefficient. S.E., standard error; Ref, reference; HGB, hemoglobin; MCV, mean corpuscular volume; RDW, red cell distribution width; WBC, white blood cell count; PDW, platelet distribution width; MPV, mean platelet volume; CRP, C-reactive protein; PCT, procalcitonin; TG, triglycerides; ALB, albumin; ALB_CRP, albumin-CRP ratio; Abdominal, infections located in the abdominal area; Pulmonary, infections located in the lungs; Urinary, infections located in the urinary tract; Other, infections located in areas not specified above.

Features	Ref	B	S.E	Wald	*P* value	OR (95% CI )
Gender	Female	− 0.520	0.371	1.965	0.161	0.594 (0.287 1.230)
Drinking	No	0.657	0.732	0.804	0.370	1.929 (0.459 8.103)
Smoking	No	2.491	1.062	5.501	0.019	12.078 (1.506 96.872)
Hypertension	No	0.432	0.401	1.158	0.282	1.54 (0.702 3.381)
Cardiovascular	No	0.468	0.617	0.575	0.448	1.596 (0.476 5.346)
Diabetes	No	0.495	0.381	1.690	0.194	1.641 (0.778 3.461)
Site	Other					
Pulmonary		− 0.446	0.680	0.431	0.512	0.60 (0.169 2.427)
Abdominal		1.500	0.659	5.183	0.023	4.480 (1.232 6.293)
Urinary		2.837	0.679	17.483	< 0.001	17.067 (4.514 4.524)
Age		0.026	0.026	1.020	0.313	1.0267 (0.976 1.081)
HGB		− 0.017	0.009	3.366	0.067	0.983 (0.965 1.001)
MCV		− 0.036	0.024	2.185	0.139	0.965 (0.920 1.012)
RDW		− 0.005	0.079	0.003	0.954	0.995 (0.853 1.162)
WBC		− 0.023	0.027	0.717	0.397	0.977 (0.926 1.031)
Neutrophil		− 0.027	0.028	0.914	0.339	0.974 (0.922 1.028)
Lymphocyte		0.438	0.367	1.424	0.233	1.550 (0.755 3.184)
Monocyte		0.020	0.601	0.001	0.974	1.020 (0.314 3.314)
Platelet		0.002	0.002	0.879	0.348	1.002 (0.998 1.006)
PDW		− 0.075	0.077	0.963	0.326	0.927 (0.798 1.078)
MPV		− 0.002	0.083	0.001	0.980	0.998 (0.848 1.175)
CRP		0.000	0.003	0.006	0.938	1.000 (0.994 1.007)
PCT		− 0.002	0.004	0.138	0.710	0.998 (0.990 1.007)
Cholesterol		− 0.187	0.241	0.605	0.437	0.829 (0.517 1.329)
TG		0.354	0.323	1.204	0.2723	1.425 (0.757 2.683)
Uric		0.000	0.001	0.044	0.833	1.000 (0.997 1.002)
ALB		0.034	0.039	0.745	0.388	1.035 (0.958 1.117)
ALB_CRP		− 0.027	0.142	0.038	0.846	0.973 (0.737 1.284)

**Table 3:** Results of multivariate logistic regression analysis in the backward stepwise elimination method. HGB, hemoglobin; Ref, reference; B: coefficient. S.E., standard error; OR, odds ratio; CI, confidence interval.

Variables	Ref	B	S.E	Wald	*P* value	OR (95% CI )
Smoking	No	− 1.590	1.122	2.007	0.157	0.204 (0.023 1.840)
Site	Other					
Pulmonary		− 0.495	0.704	0.495	0.482	0.609 (0.153 2.423)
Abdominal		1.341	0.692	3.756	0.053	3.821 (0.985 14.825)
Urinary		2.666	0.713	13.965	< 0.001	14.380 (3.552 58.207)
HGB		− 0.023	0.012	3.720	0.054	0.977 (0.954 1.000)

### LASSO analysis

**Figure 3:**
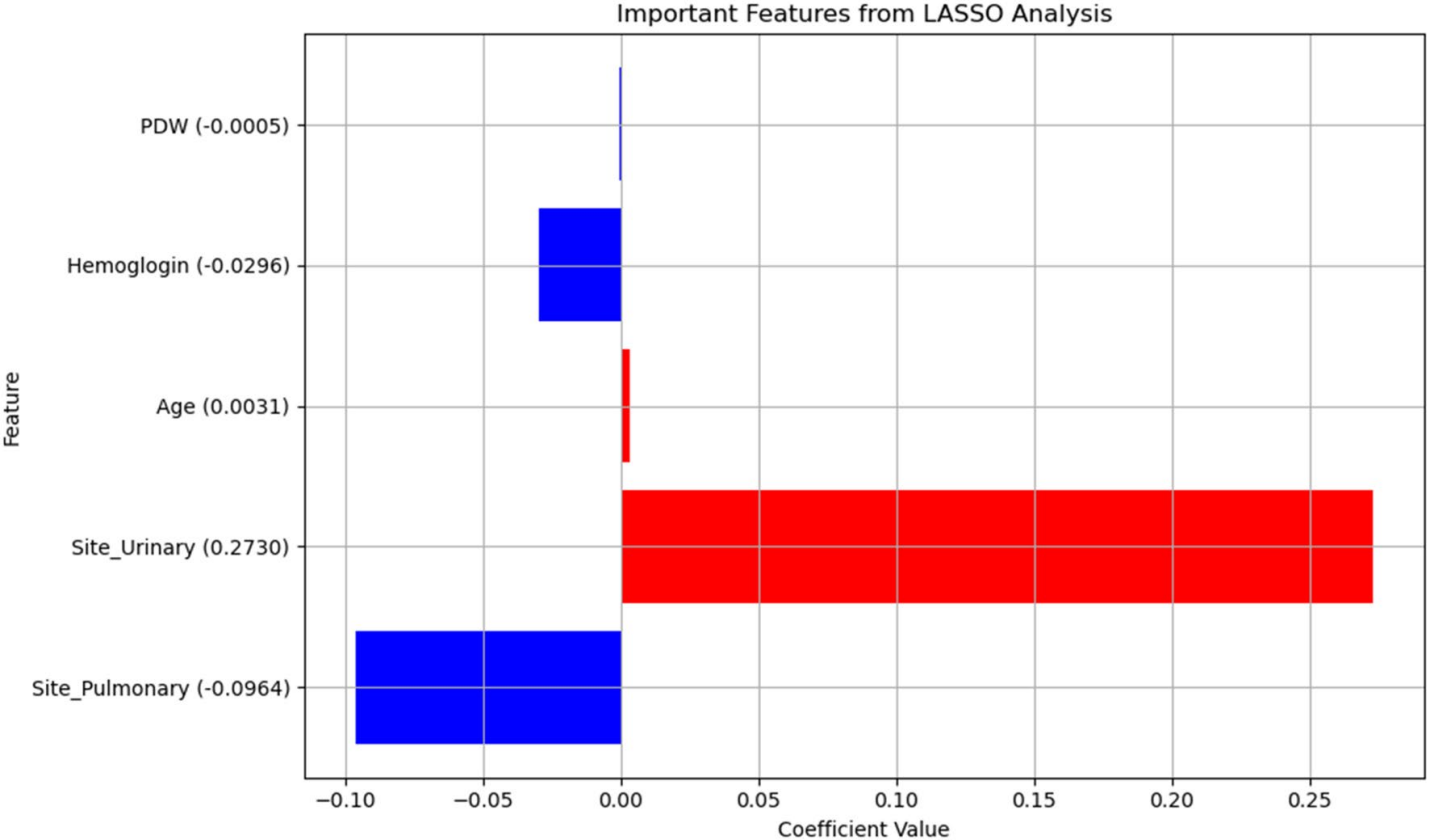
Important features identified from the LASSO analysis. The coefficients represent the impact of each feature on the prediction of the response variable. The feature 'Site_Urinary' shows a strong positive impact, while 'Site_Pulmonary' has a significant negative impact.

The LASSO analysis revealed that the positive impactful features were urinary and age, with a prominence score of 0.2730 and 0.0031. Conversely, pulmonary, HGB, PDW showed a negative influence (with negative score: 0.0964, 0.0296, 0.005, respectively) (Fig. 3).

### Random forest model analysis

**Figure 4:**
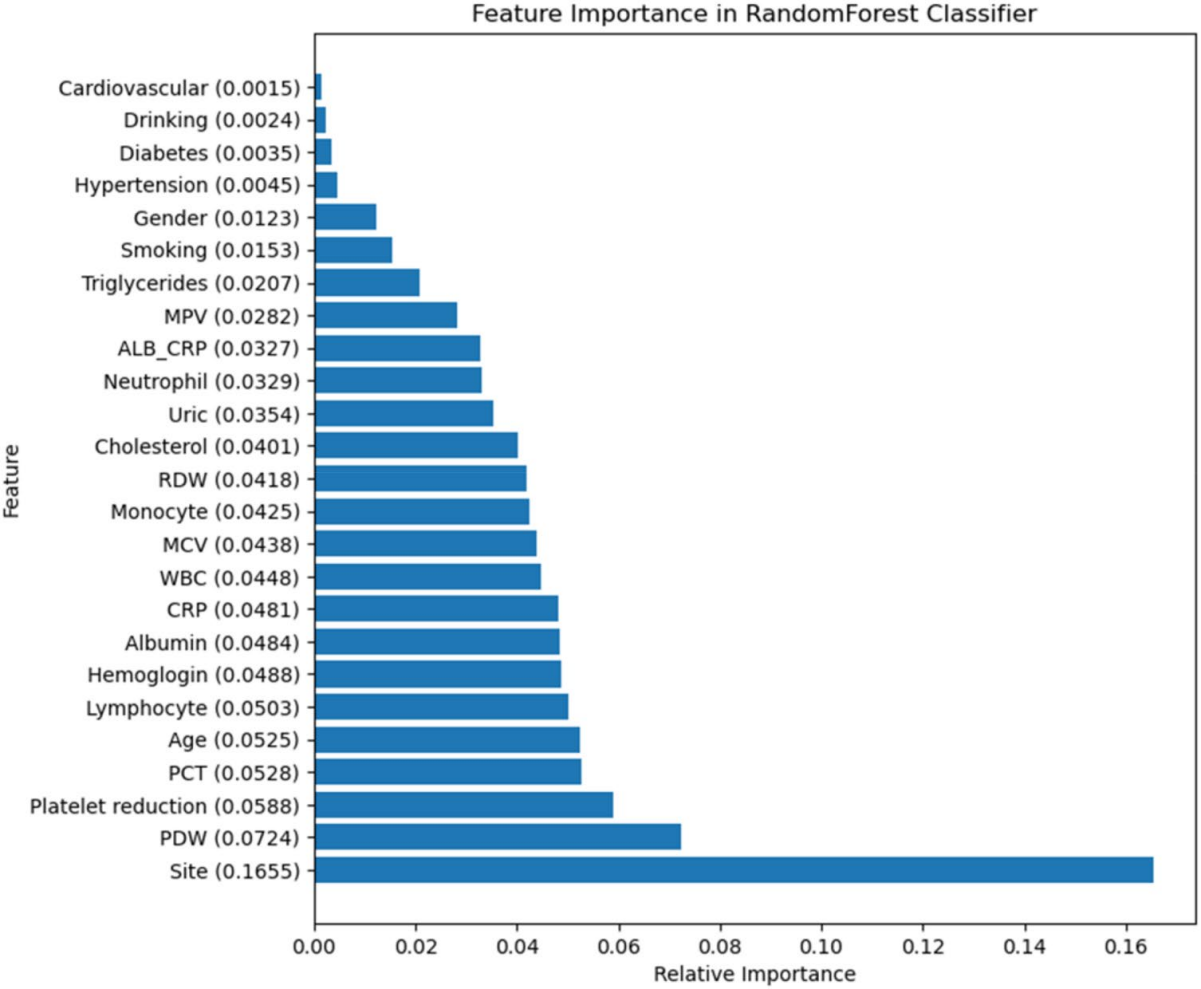
Feature importance in random forest classifier. HGB, hemoglobin; MCV, mean corpuscular volume; RDW, red cell distribution width; WBC, white blood cell count; PDW, platelet distribution width; MPV, mean platelet volume; CRP, C-reactive protein; PCT, procalcitonin; TG, triglycerides; ALB, albumin; ALB_CRP, albumin-CRP ratio.

Our analysis using the random forest model, as depicted in Fig. 4, identified critical predictors for distinguishing *E. coli* from non-*E. coli* infections among elderly sepsis patients. The site of infection emerged as the most influential feature, with a prominence score of 0.1655, followed by PDW, reduced platelet count, and PCT levels. Additionally, patient age and lymphocyte counts were significant but to a lesser degree.

**Figure 5:**
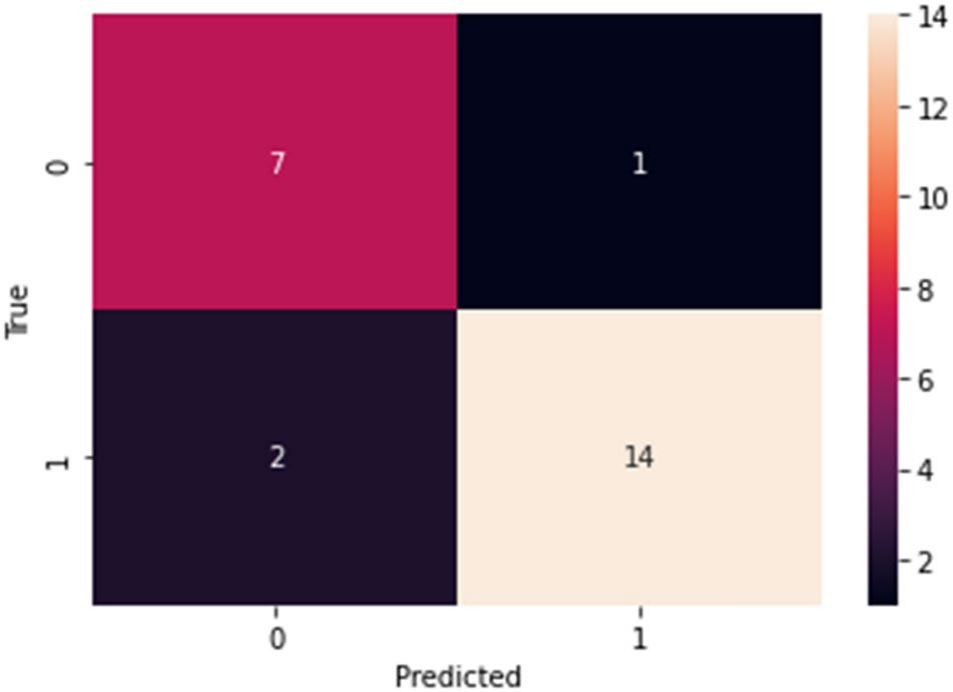
Confusion matrix for the random forest model showing the classification performance. The model correctly predicts 14 positive and 7 negative cases, while misclassifying 1 negative as positive and 2 positives as negative.

Assessing model performance, we found notable precision and recall rates in the classification of *E. coli* versus non-*E. coli* infections. The model achieved a precision of 0.78 and a recall of 0.88 for *E. coli*, which translated to an F1-score of 0.82 across 8 instances (Table 4). For non-*E. coli* infections, the precision improved to 0.93, with a recall of 0.88, resulting in an F1-score of 0.90 based on 16 instances (Table 4). Our comprehensive evaluation, reflected in the confusion matrix (Fig. 5), validates the model's predictive strength, achieving an overall accuracy of 0.88 and balanced macro-average and weighted-average F-scores of 0.86 and 0.88, respectively, across 24 samples. The model's robustness is further supported by Table 4, which summarizes the performance metrics.

**Table 4:** Performance metrics of the random forest classification model. Macro-average, average across all classes, giving each class equal weight; Weighted-average, average across all classes, weighted by support.

Class	Precision	Recall	F1-score	Support	Accuracy
*E.coli*	0.78	0.88	0.82	8	
Non-*E. coli*	0.93	0.88	0.90	16	
Macro-average	0.86	0.88	0.86	24	
Weighted-average	0.88	0.88	0.88	24	0.88

**Figure 6:**
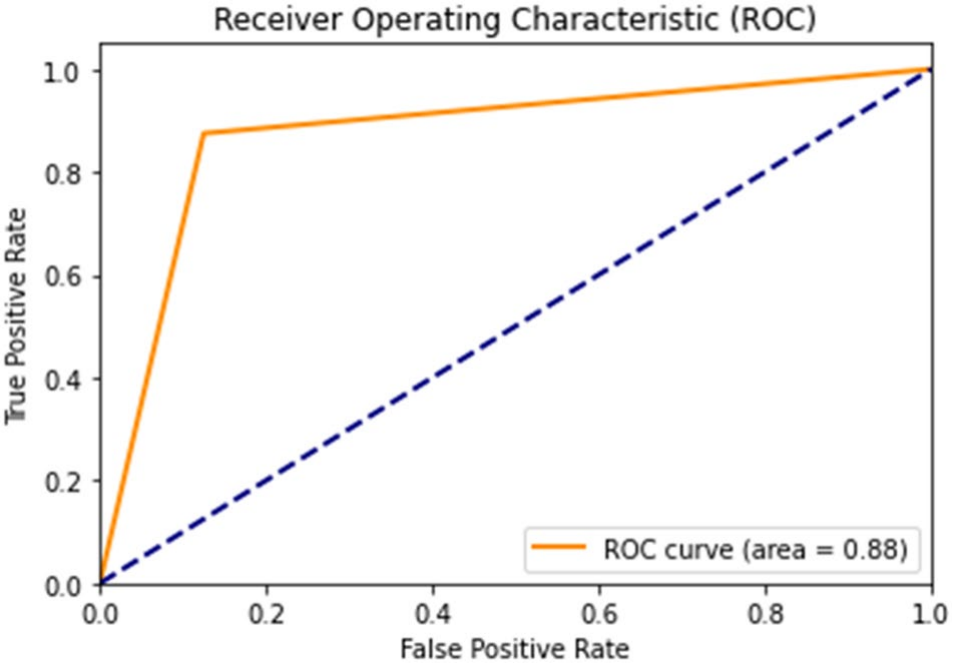
Receiver operating characteristic (ROC) curve for the random forest model. The curve has an area under the curve (AUC) of 0.88, demonstrating the model's ability to distinguish between *Escherichia coli* and non-*Escherichia coli* infections.

When analyzing the random forest model's ability to predict *E. coli* infections specifically, Table 5 shows an accuracy of 87.5%, with a precision of 93.3% and recall of 87.5%. The F1 Score stood at 90.3%, and the model demonstrated high sensitivity and specificity, both at 87.5%. The positive and negative predictive values were 93.3% and 77.8%, respectively. The receiver operating characteristic (ROC) curve, shown in Fig. 6, with an area under the curve (ROC AUC) of 88.0%, underscores the model's diagnostic efficacy. The random forest model, with its high accuracy and precision, holds significant promise for complex biological classification tasks and could be a valuable tool in the clinical management of sepsis among the elderly.

**Table 5:** Performance metrics of random forest model for predicting *Escherichia coli* infections. ROC AUC, receiver operating characteristic area under curve.

Variables	Value (%)
Accuracy	87.5
Precision	93.3
Recall	87.5
F1 Score	90.3
ROC AUC	88.0
Sensitivity	87.5
Specificity	87.5
Positive predictive value	93.3
Negative predictive value	77.8

### Five-fold cross-validation of random forest analysis

We employed the random forest model for five-fold cross-validation and assessed its performance. The results are summarized as follows: Mean accuracy: 0.697, indicating good overall performance. Standard deviation: 0.047, showing stability across validation sets. Five individual accuracy scores varied slightly (0.75, 0.71, 0.71, 0.71, 0.61), but remained within a reasonable range. These findings demonstrate the random forest model's effectiveness, with moderate accuracy and stability, laying a strong foundation for further investigation.

## Discussion

This study offers significant insights into the characteristics, biomarkers, and microbial distributions in elderly sepsis patients and evaluates predictive models for infection type differentiation. Our findings highlight the critical role of comprehensive clinical and microbiological profiling in sepsis management, especially for the elderly, who face an increased risk from various comorbidities^14^. Our analysis revealed a high prevalence of lifestyle risks and comorbidities, such as hypertension and diabetes, in our elderly cohort, further emphasizing their impact on sepsis risk. A. Komori et al.^15^ further support this by demonstrating how biomarkers such as CRP and PCT can effectively predict bacteremia in sepsis ICU patients. Their study advocates for integrating these clinical factors into predictive models to enhance sepsis outcome predictions^15^. These findings aligned with previous research, which indicated that lifestyle factors and pre-existing health conditions significantly influence sepsis outcomes in the elderly^16^.

Our results underscore the critical role of both the site of infection and specific biomarkers, including hemoglobin, PDW, reduced platelet count, and PCT, in the determination of infection types. This emphasizes the imperative for comprehensive clinical evaluations to ensure precise diagnostics. Hemoglobin levels, as a reflection of the oxygen-carrying capacity of the blood, are crucial in the assessment of sepsis severity. Low hemoglobin concentrations may indicate impaired oxygen delivery, which can exacerbate sepsis outcomes^17^. Reduced platelet count is often associated with increased severity of sepsis, as it may indicate disseminated intravascular coagulation or bone marrow suppression^18^. A low platelet count can serve as a warning sign of complications, making it a critical marker in sepsis evaluation^19^. Reduced platelet count, along with other biomarkers, provides valuable insights into the patient's immune response and infection status^20^. The significance of PDW and PCT, alongside RDW and HCT, as traditional biomarkers in the diagnosis of sepsis, is reaffirmed. Our findings resonate with the research conducted by K. Song et al.^21^, which identified RDW and HCT as significant predictors of in-hospital mortality among adult patients with E. coli-induced sepsis. This parallel underscores the importance of prompt and effective clinical assessment in improving sepsis patient outcomes.

The random forest model's success in distinguishing *E. coli* from non-*E. coli* infections underscores machine learning's potential to enhance diagnostic accuracy. This parallels the findings of Jeng et al., who used similar techniques to predict recurrent urinary tract infections caused by *E. coli*^22^. The identification of key features such as the site of urinary and pulmonary infections, PDW, reduced platelet count, and PCT as crucial predictors further supports for the amalgamation of clinical and laboratory data in constructing predictive models. Previous research by J. Shi et al.^9^ highlighted the pivotal role of PCT as a particularly effective biomarker for discerning sepsis patients. Additionally, findings from a study by M. Su et al.^23^ demonstrated that among 17 statistically significant biomarkers, PCT exhibited the highest AUC for diagnosing urosepsis. The integration of these models has the potential to substantially enhance the speed and accuracy of sepsis diagnosis, facilitating more timely and precise interventions.

Understanding the site of infection is essential for formulating clinical strategies for managing elderly sepsis^24^. Our study revealed that urinary tract infections were the most common infectious site among elderly sepsis patients (32%), followed by pulmonary infections (30%), abdominal infections (20%), and other sites (18%). This finding aligns with prior research conducted by J. Doua et al.^25^, which also identified the urinary tract as the primary source of infection (62.9%), followed by intraabdominal infections (20.4%), other infections (14.2%), and respiratory tract infections (2.5%). Urinary and pulmonary infections are particularly critical in the context of sepsis, particularly in distinguishing between *E. coli* and non-*E. coli* infections. *E. coli*, a common Gram-negative bacterium in the gastrointestinal tract, frequently causes urinary tract infections^2,26^. Our research uncovers a multifaceted microbial environment in sepsis, predominantly characterized by Gram-negative bacteria, especially *E. coli*, along with other non-*E. coli* bacteria. A previous study^9^ showed that *E. coli* (40.0%) was the predominant bacterial finding in COVID-19 sepsis patients. This complexity underscores the urgent need for a broad-spectrum empirical diagnostic approach, which is particularly crucial for managing sepsis in vulnerable groups, such as the elderly. Similarly, *Klebsiella pneumoniae* is a leading cause of pulmonary infections, such as pneumonia^27,28^. In sepsis, the infection site is crucial, as it acts as an entry point for pathogens and triggers systemic inflammatory responses^29^. This can lead to severe sepsis or septic shock, particularly when *E. coli* is involved, given its virulence factors and ability to evade host immune responses^30^.

### Limitations and future directions

Our study has limitations, including its sample size and single-center design, which may affect the generalizability of the results. Future research should focus on multicenter studies with larger, more diverse populations to enhance the robustness and applicability of the findings. Further exploration of the mechanisms behind identified associations and the integration of genomic and proteomic data into the machine learning model could provide deeper insights into the pathophysiology of sepsis in elderly patients^31^.

## Methods and materials

### Study design and participants

This retrospective study was conducted at the Department of Clinical Laboratory, Fuding Hospital, Fujian University of Traditional Chinese Medicine. Medical records of 119 elderly patients (aged ≥ 60 years) diagnosed with sepsis from January to December 2022, were reviewed. Patients were divided into two groups: *E. coli* infections (case group, n = 57) and non-*E. coli* infections (control group, n = 62). Inclusion criteria specified individuals over 60 years with solitary bacterial growth in blood cultures from sepsis patients. Exclusion criteria encompassed subjects with significant heart or liver function abnormalities, a history of tumors or coagulation dysfunction, pregnancy or breastfeeding, and recent trauma or surgery.

The study protocol obtained approval from the Medical Ethics Committee of Fuding Hospital, Fujian University of Traditional Chinese Medicine, with the ethical approval number Fuding Hospital 2,022,325. All methods were performed in accordance with the relevant guidelines and regulations. Due to its retrospective nature, the study was exempted from requiring written informed consent by the Medical Ethics Committee of Fuding Hospital, Fujian University of Traditional Chinese Medicine.

### Bacterial identification and detection of biomarkers

Peripheral venous blood samples, collected from patients prior to antibiotic therapy initiation using sterile techniques to minimize contamination. Samples were immediately inoculated into Bactec culture vials to facilitate aerobic and anaerobic bacterial growth. The vials were then placed in a Bactec incubator (BD Diagnostics, Franklin Lakes, NJ, USA) and monitored for bacterial growth. Only bacterial isolates meeting predefined pathogenicity criteria were analyzed further, ensuring the findings' relevance to clinical sepsis.

For the purpose of this study, isolated pathogens were classified into two primary categories: *E. coli* and non-*E. coli* bacteria. Within these categories, bacteria were further organized into distinct phylogenetic groups to facilitate a detailed analysis of microbial diversity in sepsis. These groups included *E. coli, Klebsiella pneumoniae, Staphylococcus *spp.*, Streptococcus *spp.*,* and *Enterococcus *spp. To accommodate the identification of less common pathogens, a novel sixth category was established. This category included pathogens that did not fit into the aforementioned groups but were identified as clinically significant based on specific pathogenic criteria.

Further classification within the *Staphylococcus* and *Enterococcus* genera was conducted to provide insight into the specific species contributing to sepsis in the elderly population. The *Staphylococcus *spp. group comprised *Staphylococcus aureus*, *Staphylococcus epidermidi*s, *Staphylococcus saprophyticus*, and *Staphylococcus haemolyticus*, while the *Enterococcus *spp. group included *Enterococcus faecalis*, *Enterococcus faecium*, *Enterococcus gallinarum*, and *Enterococcus avium*. This detailed categorization was essential for understanding the microbiological landscape of sepsis in the study population.

Clinical data, meticulously extracted from electronic medical records, included demographic details, lifestyle behaviors (such as smoking and drinking habits), comorbidities (including hypertension, cardiovascular diseases, and diabetes), and a comprehensive set of laboratory measurements. These measurements included HGB, MCV, RDW, WBC, neutrophil, lymphocyte, monocyte, platelet counts, PDW, MPV, CRP, PCT, cholesterol, triglycerides, uric acid, albumin, and the albumin-CRP (ALB-CRP) ratio. The infection site for each patient was carefully recorded, encompassing pulmonary, abdominal, urinary, and other locations. All biomarkers were evaluated within the initial 24 h after admission.

Serum PCT levels were accurately measured using the Cobas e411/E601 systems (Roche Diagnostics, Mannheim, Germany), renowned for their precision in diagnostic assays. CRP levels were determined with the Dimension Vista 1500 Intelligent Lab system (Siemens Healthcare GmbH, Erlangen, Germany), adhering strictly to the manufacturer’s guidelines to ensure accuracy. The Beckman Coulter AU 5800, a state-of-the-art fully automated clinical chemistry analyzer, was employed for the quantification of cholesterol, triglycerides, uric acid, albumin, and the ALB-CRP ratio, facilitating a comprehensive lipid and protein profile assessment.

Furthermore, the Sysmex XN-9000 hematology analyzer (Sysmex Corporation, Kobe, Japan), a cutting-edge instrument, was utilized for conducting a complete blood cell count, including measurements of HGB, MCV, RDW, WBC, neutrophil, lymphocyte, monocyte, platelet counts, PDW, and MPV. The ALB-CRP ratio, a novel marker of inflammation and nutritional status, was calculated by dividing the albumin value by the CRP level, offering additional insights into the patient's health status and the systemic response to infection.

### Statistical analysis

Statistical analysis was conducted using the Statistical Package for Social Sciences (SPSS) Version 22.0 (IBM Corp., Armonk, NY, USA), GraphPad Prism 8.0 (GraphPad software, San Diego California USA, www. GraphPad. com), the *R* package “CBCgrps”^13^, and Python 3.7. This method enabled thorough data analysis and the application of machine learning, ensuring the reliability and reproducibility of our results. The Shapiro–Wilk test assessed variable distribution patterns in *E. coli* and non-*E. coli* groups, yielding median values and interquartile ranges (IQRs). For the analysis of categorical data or proportions across the two groups, either the Chi-square test or Fisher’s exact test was utilized, depending on the data's suitability. Baseline characteristics of study participants were summarized using descriptive statistics. Continuous variables were presented as either median (Q1, Q3) for those not following a normal distribution or as mean ± standard deviation (SD) for data with a normal distribution. For categorical variables, frequencies and percentages were reported. The comparison of continuous variables between groups was conducted using either the Mann–Whitney U test or the Student's *t* test, based on the distribution characteristics of the data.

### Logistic regression analysis

Univariate and multivariate logistic regression analyses were carried out to ascertain factors linked with the bacterial infection type, specifically distinguishing between *E. coli* and non-*E. coli* infections. Odds ratios (ORs) with 95% confidence intervals (CIs) were computed to quantify the strength and direction of associations. Variables demonstrating an association with the outcome in the univariate logistic regression analysis (*p* value < 0.20)^32^ were subsequently incorporated into the multivariate logistic regression model using the backward stepwise elimination method to adjust for potential confounders and to identify independent predictors of *E. coli* infection subtype. Variables with a *P*-value higher than 0.05 were omitted from the multivariate logistic regression model. This methodological rigor underscored our commitment to unveiling statistically significant and clinically relevant determinants that might affect the risk of particular bacterial infections among elderly sepsis patients.

### LASSO regression analysis

Least absolute shrinkage and selection operator (LASSO) was utilized to identify key predictors. LASSO enhances prediction accuracy and model interpretability by constructing a penalty function that reduces model complexity. By shrinking some regression coefficients toward zero based on their absolute size, LASSO performs variable selection and simplifies the model. This technique is particularly valuable for datasets with multicollinearity or when the number of predictors exceeds the number of observations.

### Random forest model

A random forest model was developed to distinguish between *E. coli* and non-*E. coli* infections. The model's performance was evaluated using accuracy, precision, recall, F1 score, and the area under the ROC curve. Feature importance was assessed to identify the most significant predictors.

The Random Forest model's efficacy in differentiating *E. coli* from non-*E. coli* infections was validated using a split-sample technique, dividing data into an 80% training set and a 20% testing set. Evaluation metrics derived from the testing set underscored the model’s predictive accuracy and its potential utility in clinical settings.

## Conclusions

This study underscores the complexity of sepsis in the elderly, driven by clinical, laboratory, and microbiological factors. The significant correlations between clinical biomarkers, the diversity of microbial etiologies, and the effectiveness of predictive modeling in our analysis all point towards the need for a multifaceted approach to sepsis management. Future research should focus on refining these predictive models and exploring the integration of novel biomarkers to further enhance the early diagnosis of sepsis, ultimately improving outcomes in this vulnerable population.

## Data Availability

Te datasets used and/or analysed during the current study available from the corresponding author on reasonable request.
